# High-Sensitivity Cardiac Troponin-I Is Elevated in Patients with Rheumatoid Arthritis, Independent of Cardiovascular Risk Factors and Inflammation

**DOI:** 10.1371/journal.pone.0038930

**Published:** 2012-06-28

**Authors:** William S. Bradham, Aihua Bian, Annette Oeser, Tebeb Gebretsadik, Ayumi Shintani, Joseph Solus, Joel Estis, Quynh Anh Lu, John Todd, Paolo Raggi, C. Michael Stein

**Affiliations:** 1 Division of Cardiology, Department of Medicine, Vanderbilt University Medical Center, Nashville, Tennessee, United States of America; 2 Division of Clinical Pharmacology, Department of Medicine, Vanderbilt University Medical Center, Nashville, Tennessee, United States of America; 3 Department of Biostatistics, Vanderbilt University Medical Center, Nashville, Tennessee, United States of America; 4 Department of Molecular Physiology and Biophysics, Vanderbilt University Medical Center, Nashville, Tennessee, United States of America; 5 Division of Cardiology, Emory University, Atlanta, Georgia, United States of America; 6 Singulex Inc., Alameda, California, United States of America; University of Otago, New Zealand

## Abstract

**Objectives:**

We examined the hypothesis that cardiac-specific troponin-I (cTn-I), a biomarker of myocardial injury, is elevated in patients with rheumatoid arthritis (RA).

**Background:**

RA patients have an increased incidence of heart failure (HF). Chronic myocardial injury in RA may be a mechanism for the development of HF.

**Methods:**

We compared cTn-I concentrations measured by high-sensitivity immunoassay in 164 patients with RA and 90 controls, excluding prior or active heart failure. We examined the relationship between cTn-I concentrations and cardiovascular risk factors, inflammation, and coronary artery calcium score (CACS), a measure of coronary atherosclerosis.

**Results:**

cTn-I concentrations were 49% higher in patients with RA (median 1.15 pg/mL [IQR 0.73–1.92] than controls (0.77 pg/mL [0.49–1.28](P<0.001). The difference remained statistically significant after adjustment for demographic characteristics (P = 0.002), further adjustment for cardiovascular (CV) risk factors (P = 0.004), inflammatory markers (P = 0.008), and in a comprehensive model of CV risk factors and inflammatory markers (P = 0.03). In patients with RA, cTn-I concentrations were positively correlated with age (rho = 0.359), Framingham risk score (FRS) (rho = 0.366), and systolic blood pressure (rho = 0.248 (all P values ≤0.001)), but not with measures of inflammation or RA drug therapies. cTn-I was significantly correlated with CACS in RA in univariate analysis, but not after adjustment for age, race, sex and FRS (P = 0.79). Further model adjustments for renal function and coronary artery disease confirmed the significance of the findings.

**Conclusion:**

High-sensitivity cTn-I concentrations are elevated in patients with RA without heart failure, independent of cardiovascular risk profile and inflammatory markers. Elevated troponin concentrations in RA may indicate subclinical, indolent myocardial injury.

## Introduction

Patients with rheumatoid arthritis (RA) experience premature mortality that is largely due to cardiovascular disease [1], [2]. Cardiovascular mortality in RA is 50–60% higher compared to the general population, and there is increased prevalence of ischemic heart disease [3]–[6], There is a markedly higher prevalence of congestive heart failure (CHF) in RA patients. For example, in a longitudinal, population-based study, the 30-year cumulative incidence of CHF in RA was 37.1% compared to 27.7% in controls, and CHF, rather than ischemic heart disease, accounted for the majority of the increase in cardiovascular mortality in RA [7].

We have previously reported increased concentrations of N-terminal pro-brain natriuretic peptide (NT-proBNP) in patients with RA [8]. NT-proBNP is released by myocytes in response to myocardial stretch, and subtle increases may reflect subclinical myocardial dysfunction [9]. Cardiac magnetic resonance imaging has demonstrated decreased left ventricular mass in patients with RA [10]. Thus, RA may predispose to the development of heart failure through a pathophysiology distinct from that of ischemic heart disease; and chronic, indolent myocyte loss may be a central process.

Cardiac troponins (cTn) are components of the cardiomyocyte contractile apparatus, and circulating concentrations are elevated in the setting of myocardial injury, such as acute coronary syndromes (ACS) [11]. High-sensitivity (hs) cTn assays allow measurement of troponin concentrations below conventional levels of detection and have revealed a spectrum of circulating cTn concentrations spanning low and high levels in both healthy subjects and in patients with overt cardiovascular disease. Low levels of troponin elevation, below threshold levels used to diagnose ACS, are associated with increased CV mortality, both in patients with diagnosed ischemic heart disease and in the general population [12], [13].

There is no information about cardiac troponin concentrations in patients with RA. We hypothesized that concentrations high sensitivity cardiac troponin (hs-cTn), a marker of myocyte injury, may be elevated early in the disease process, before the development of clinical heart failure.

## Methods

### Ethics Statement

The study was approved by the Institutional Review Board (IRB) of Vanderbilt University Medical Center, and all subjects were provided written informed consent.

### Patients and Control Subjects

Subjects were participants in an ongoing study of cardiovascular risk in RA, and detailed methods have been described previously [14], [15]. One hundred and sixty four eligible patients >18 years of age who met the American College of Rheumatology classification for RA and 90 control subjects without RA were studied. Individuals with heart failure, defined as current heart failure requiring treatment, or fulfillment of at least 2 of three following criteria; a previous history of heart failure, use of digoxin, or use of a diuretic, were excluded from the study.

Patients were recruited from an RA registry, by referral from area rheumatologists, and by local advertisement. Control subjects were recruited from among the patient’s acquaintances, by local advertisement, and from a database of volunteers maintained by the Vanderbilt Clinical Research Center.

### Data Collection

Clinical and demographic information was acquired via structured interview, questionnaires, physical examination, laboratory tests, electron beam computed tomography (CT), and review of medical records. Current and historic medication use was determined from information provided by patients, and from review of medical records. Blood pressure was recorded as the average of 2 measurements obtained 5 minutes apart after the subject had rested in a supine position for at least 10 minutes. Subjects were considered hypertensive if they were taking antihypertensive agents, or if measured systolic blood pressure was greater than 140 mm Hg and/or diastolic pressure was greater than 90 mm Hg. RA disease activity was assessed using the Disease Activity Score based on evaluation of 28 joints (DAS28). The DAS28 is a validated composite index including a 28 joint count for tenderness and swelling, erythrocyte sedimentation rate (ESR), and the patient’s overall assessment of wellbeing [16]. The Framingham risk score (FRS), a composite score of traditional risk factors that includes blood pressure, smoking status, age, and sex, but not diabetes mellitus, was calculated.

### Laboratory Tests

Blood was collected after an overnight fast for determination of complete blood count, serum creatinine, total cholesterol, high-density lipoprotein (HDL) cholesterol, triglycerides, homocysteine, and low-density (LDL) cholesterol at the Vanderbilt University Medical Center clinical laboratory. In patients with RA, CRP and Westergren ESR were measured by the hospital laboratory, and medical records reviewed to determine the presence or absence of rheumatoid factor (RF). Before 2003, the laboratory did not use a high-sensitivity CRP assay, and low concentrations were reported as <3 mg/L. Thus, in 40 patients with CRP reported as <3 mg/L, concentrations were re-measured by ELISA (Millipore), and the resulting data were used in the current study analysis. High sensitivity cardiac specific troponin-I (hs-cTn-I) was assayed using the Erenna^R^ System (Singulex Corp, Alameda, CA) by technicians blinded to the clinical data. The limit of detection and the 10% coefficient of variation were 0.1 pg/mL and 0.7 pg/mL, respectively. TNFa, interleukin-6 (IL-6), and NT-pro-BNP concentrations were measured by multiplex enzyme-linked immunosorbent assay (Linco Research/Millipore, St. Louis, MO).

### Coronary Artery Calcification

All subjects underwent tomographic imaging with a c-150 scanner (GE/Imatron, South San Francisco, CA) as described previously [14]. Scans were evaluated by a single investigator blinded to patient clinical status. The degree of calcified plaque was calculated as described previously; the sum of partial scores of all coronary artery lesions provided an overall coronary artery calcium score (CACS) for each patient [13].

### Statistical Analysis

Descriptive statistics were calculated as the median with the interquartile range (median [IQR]) or mean ±SD) according to the distribution of the continuous variables. Wilcoxon rank-sum tests were used to compare continuous variables. Person Chi-square tests were used to compare categorical variables. We assessed the correlation between hs-cTn-I concentration and cardiovascular risk factors (body mass index (BMI), high density lipoprotein (HDL) and low density lipoprotein (LDL) cholesterol, triglycerides (TG), diabetic status, homocysteine, lipoprotein(a), inflammation markers (CRP, IL-6 and TNFα) with Spearman’s rank correlation test separately for patients with RA and control subjects. To assess the independent association between RA status and hs-cTn-I concentration, we used 4 multivariable linear regression models with a priori defined covariates. Those models adjusted for:

#### Model 1

Age, race, and sex (base model)

#### Model 2

Base model and cardiovascular risk factors (body mass index (BMI), high density lipoprotein (HDL) and low density lipoprotein (LDL) cholesterol, triglycerides (TG), diabetic status, homocysteine, lipoprotein(a), current smoking status, and hypertension (HTN))

#### Model 3

Base model and markers of inflammation (C reactive protein (CRP), interleukin-6 (IL-6), and tumor necrosis factor-alpha (TNFa) and

#### Model 4

Base model, cardiovascular risk factors (as in Model 2), markers of inflammation (as in Model 3), and NT-proBNP.

**Table 1:** Clinical Characteristics of Study Subjects.

Characteristic	RA (n = 164)			Control (n = 90)		P-Value
**Demographics (mean±SD)**						
Age (years)		54.0±11.8	52.9±11.3		0.422	
Female Sex, no. (%)		112 (68%)	56 (62%)		0.328	
Caucasian, no. (%)		147 (90%)	76 (84%)		0.227	
Non-smoker, no. (%)		39 (24%)	8 (9%)		0.003	
**Disease Activity Score (DAS28)**		3.9 (2.6–4.9)	-		-	
**Disease Duration (years)**		3 (2–18)	-		-	
**Cardiovascular Risk Factor**						
Hypertension, no. (%)		85 (52%)		35 (39%)		0.048
Systolic BP, mmHg (mean±SD)		132.8±20.2		129.1±17.5		0.133
Diastolic BP, mmHg (mean±SD)		74.81±10.89		72.74±8.82		0.185
CVD or coronary procedure, no. (%)		22 (13%)		8 (9%)		0.285
Diabetic, no. (%)		18 (11%)		4 (4%)		0.077
On NSAIDS, no. (%)		54 (33%)		36 (40%)		0.26
BMI, kg/m^2^ (mean±SD)		29.14±6.63		28.54±5.88		0.543
**CACS (Agatston Units) (median, IQR)**		2.7 (0.0–150.4)		0.0 (0.0–19.2)		0.021
**RA treatment regimens**						
Current corticosteroid use, no. (%)	88 (54%)			-		-
Current methotrexate use, no. (%)	118 (72%)			-		-
Current antimalarial use, no. (%)	42(26%)			-		-
Current anti-TNF agent use, no. (%)	34 (21%)			-		-
**Laboratory Measurements (median, IQR)**						
HDL (mg/dL)	43.0 (37.0–54.0)			45.0 (38.0–54.0)		0.705
LDL (mg/dL)	111.0 (88.5–135.5)			121.0 (104.0–140.0)		0.031
Homocysteine (µmol/L)	2.317 (2.083–2.477)			2.104 (1.978–2.270)		<0.001
Creatinine (mg/dL)	0.800 (0.700–0.900)			0.800 (0.700–0.900)		0.408
TNFa (pg/mL)	5.41 (2.72–11.00)			3.38 (2.35–4.67)		<0.001
IL-6 (pg/mL)	13.81 (4.41–42.36)			4.25 (1.15–18.27)		<0.001
CRP (mg/L)	4.00 (1.19–11.00)			0.55 (0.21–1.53)		<0.001
NT-BNP (pg/mL)	89.7 (28.7–232.3)			18.0 (3.2–39.0)		<0.001
hs-cTn-I (pg/mL)	1.150 (0.730–1.922)			0.770 (0.492–1.277)		<0.001

P values were determined by Wilcoxon’s rank sum test or chi-square test.

Among the RA group, the association between hs-cTn-I concentrations and CACS was examined using the proportional odds model with CACS as the dependent variable, and were adjusted for age, race, sex, and FRS. Concentrations of hs-cTn-I, triglycerides, homocysteine, CRP, TNF-α, and IL-6 were natural logarithm-transformed to improved normality. All calculations were performed using R version 2.10.0 (http://www.r-project.org) and two-sided P values less than 0.05 were considered statistically significant.

### Sensitivity Analysis for Possible Confounders

Because cardiac troponin is renally cleared, the results of the study could be confounded in patients with poor renal function. To account for renal function, the statistical models were analyzed by including the estimated glomerular filtration rate (Modification of Diet in Renal Disease calculation) in the model. Further, to control for the presence of known cardiovascular disease in the study subjects, the statistical models were re-analyzed, excluding patients with a diagnosed cardiovascular disease (22 RA patients, 8 control patients excluded).

## Results

### Patient Characteristics

Patients with RA (n = 164) and control subjects (n = 90) were of similar age and had a similar sex distribution (Table 1). As reported previously in this cohort, NT-BNP, CRP, TNFa, IL-6, homocysteine, and CACS were significantly higher in RA than in control subjects [9], [14], [15]. A history of ischemic cardiovascular disease (stroke, myocardial infarction, angina) or a coronary procedure (coronary artery bypass graft or angioplasty) was present in 13% of patients with RA and 9% of controls (P = 0.29).

### High Sensitivity cTn-I Concentrations and Clinical Variables in RA Patients and Controls

**Figure 1:**
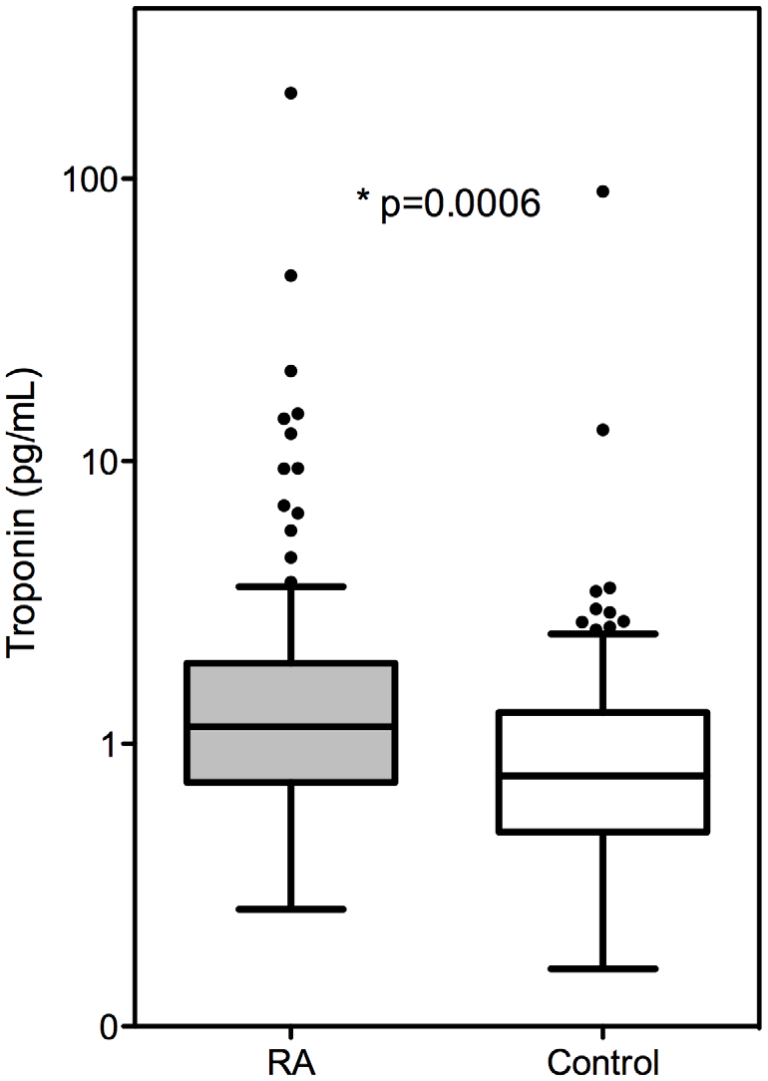
Unadjusted Troponin Concentrations. Boxplot of troponin concentrations (pg/mL) for RA subjects and controls, demonstrating elevated troponin concentrations in RA patients compared to controls (p = 0.0006). Presented as 25th quartile, median, 75th quartile. Fences drawn to nearest value not exceeding 1.5 interquartile range. Observations beyond fences are plotted.

**Figure 2:**
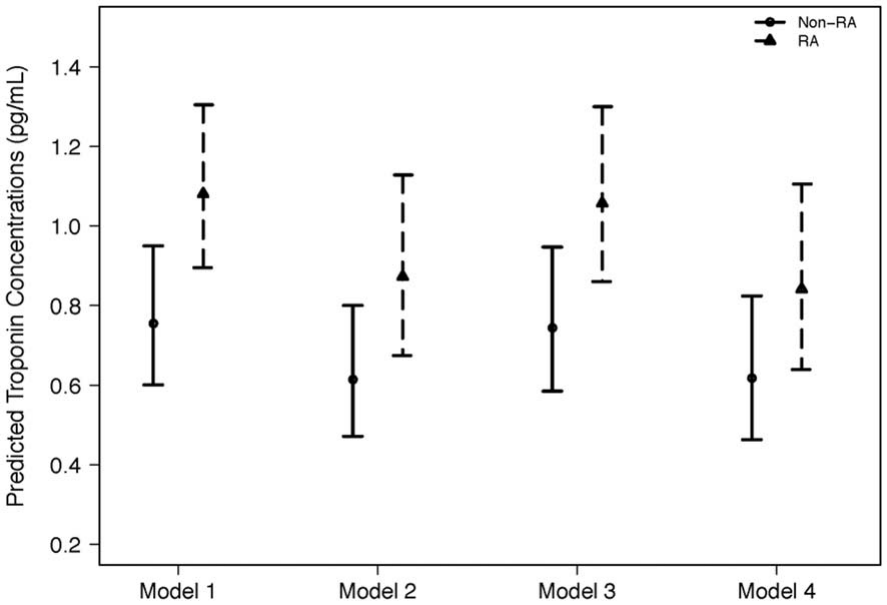
Multivariable Linear Regression Troponin Models for RA Patients vs Controls. Model 1: Adjusted for patient age, race, gender (p = 0.002)**;** Model 2: Adjusted for age, race, gender, and cardiovascular risk factors (p = 0.004); Model 3: Adjusted for age, race, gender and markers of inflammation (p = 0.008); Model 4: Adjusted for age, race, gender, cardiovascular risk factors, markers of inflammation, and NT-proBNP (p = 0.03).

High sensitivity cTn-I concentrations were significantly higher in patients with RA (median 1.15 pg/mL [IQR 0.73–1.92] than controls (0.77 pg/mL [0.49–1.28](P<0.001) (Figure 1). Hs cTn-I remained significantly higher in RA patients after adjustment for **age, race, and sex** (p = 0.002)(Figure 2- **Model 1**); age, **race, sex and cardiovascular risk factors** (p = 0.004)(Figure 2- **Model 2**); **age, race, sex, and markers of inflammation** (p = 0.008)(Figure 2- **Model 3**); and **age, race, sex, CV risk factors, markers of inflammation, and NT-proBNP** (p = 0.029)(Figure 2- **Model 4**). Rheumatoid factor was measured in 157 patients, and hs-cTn-I concentrations were not significantly different among RA patients in whom rheumatoid factor was present (1.17 [0.73–1.99] pg/ml, n = 112 (71%)), or absent (1.09 [0.73–1.41] pg/ml, n = 45 (29%))(p = 0.28).

### Sensitivity Analysis

Sensitivity analyses were performed adjusting for renal function (MDRD) and excluding study subjects with previous CV disease. The finding that hs-cTn-I was higher in RA patients compared to controls remained statistically significant in models 1–4 (all P values <0.05).

### Association between hs-cTn-I Concentrations and Clinical Variables

In RA, hs-cTn-I concentrations were positively correlated with age, systolic BP, CACS and NT-proBNP; and in control subjects with systolic BP, serum creatinine, and CACS, but not with age or NT-proBNP (Table 2). There was no significant difference in hs-cTn-I concentrations among RA patients who were or were not currently receiving methotrexate, anti-TNF drugs, hydroxychloroquine, and corticosteroids (Table 3).

**Table 2:** Correlation between hs-cTn-I and clinical variables in patients with RA and controls.

	RA Patients (n = 164)		Controls (n = 90)	
Variable	rho	P*	rho	P*
Age	0.359	<0.001	0.172	0.104
Body mass index	−0.142	0.069	0.026	0.806
Systolic blood pressure	0.248	0.001	0.297	0.005
Coronary calcium score	0.330	<0.001	0.298	0.005
HDL cholesterol	0.066	0.402	−0.056	0.605
LDL cholesterol	0.174	0.027	0.154	0.15
Homocysteine	0.166	0.035	0.088	0.409
Creatinine	0.145	0.064	0.306	0.003
TNFa	0.148	0.061	−0.106	0.321
IL-6	0.093	0.243	−0.144	0.188
C-reactive protein	−0.086	0.275	0.166	0.119
NT-proBNP	0.255	0.001	0.062	0.567
Disease Duration	0.197	0.012	N/A	N/A
DAS28	0.037	0.638	N/A	N/A
Erythrocyte sedimentation rate	−0.014	0.863	N/A	N/A

* P values by Spearman’s correlation coefficient test.

**Table 3:** Concentrations of hs-cTn-I in patients with RA receiving different drugs (n = 164).

Current Drug Therapy	n	hs-cTn-I, *p*g/mL	P value*
On methotrexate	118	1.170 (0.732–1.802)	0.897
Not on methotrexate	46	1.08 (0.723–1.997)	
On anti-TNF drug	34	0.980 (0.750–1.627)	0.734
Not on anti-TNF drug	130	1.175 (0.723–1.952	
On hydroxychloroquine	42	1.095 (0.730–1.655)	0.428
Not on hydroxychloroquine	122	1.170 (0.732–1.927	
On corticosteroids	88	1.175 (0.737–1.942)	0.343
Not on corticosteroids	76	1.115 (0.728–1.787)	

* Wilcoxon’s rank sum test.

### High Sensitivity cTn-I and Coronary Artery Calcification Score (CACS)

Although CACS was significantly correlated with hs-cTn-I concentrations in both patients with RA (rho = 0.33, P<0.001) and control subjects (rho = 0.24, P = 0.005), there were no statistically significant associations after multivariable model adjustment for age, race, sex and FRS in both RA (P = 0.79) and control subjects (P = 0.43).

## Discussion

The major finding of this study was that concentrations of high sensitivity cardiac troponin-I, a biomarker of cardiac injury, were higher in patients with RA compared to controls. This difference in troponin-I concentrations between patients with RA and controls remained statistically significant after adjustment for demographic variables, inflammation, renal function, coronary artery disease, and cardiovascular risk factors.

As the preferred biomarkers of myocardial necrosis in patients suspected of having acute coronary syndrome (ACS), elevated cTn concentrations have almost complete specificity for myocardial tissue. Consensus guidelines recommend that cardiac troponin concentrations corresponding to the 99^th^ percentile of a healthy population (commonly 0.03 µg/L or 30 pg/mL) be used as the threshold for defining an abnormal elevation [17]–[20]. More recently, high-sensitivity troponin assays allow measurement of troponin with detection limits 10-fold lower than conventional assays, and are thus capable of identifying subclinical myocardial injury and CV risk [12]. Low level hs-cTn elevation, is associated with increased risk of heart failure and cardiovascular death in patients with stable coronary artery disease [13]. Similarly, in several community-based studies, the troponin concentration detected was predictive of long-term cardiovascular outcomes [21], [22]. For example, in the Atherosclerosis Risk in Communities (ARIC) cohort, cTn concentrations of 6–8 pg/mL were associated with significantly increased risk for coronary heart disease, heart failure, and mortality [22].

Patients with RA have shorter life expectancy, more ischemic heart disease and heart failure, and higher cardiovascular mortality compared to the general population [23], [24], [25]. The increased risk of heart failure in patients with RA is not explained by traditional cardiovascular risk factors, or solely by the presence of ischemic heart disease [8], [26], [27]. Interestingly, a recent cardiac MRI study reported lower myocardial mass in patients with RA compared to age matched controls [10], suggesting chronic myocardial injury or myocyte loss as an underlying problem in RA. Our finding of increased circulating hs-cTn would be concordant with that hypothesis.

The mechanistic explanation for elevated troponin concentrations in RA is not known. There are several proposed myocardial mechanisms for cTn release, including subendocardial ischemia leading to myocyte necrosis, cardiomyocyte injury from inflammatory cytokines, hibernating myocardium, and apoptosis [28]–[32]. We found no significant association between hs-cTn-I levels and inflammatory cytokines, acute phase reactants, or a disease activity score (Table 2). This suggests that active inflammation may not be the primary driver of troponin elevation in RA.

Viable cardiomyocytes, without necrosis, can release cTn as an intact protein by a stretch-related mechanism mediated by integrins [33]. Thus, underlying hemodynamic stress leading to increased myocardial stretch may play a role in the elevated troponin concentrations in RA. The mechanism for BNP release from myocytes is also hypothesized to be stretch related. Indeed, we previously reported that NT-proBNP concentrations are elevated in patients with RA [9], and in the present study, hs-cTn-I and NT-proBNP concentrations were significantly correlated. However, the concentrations of hs-cTn-I remained significantly higher in patients with RA than controls, even when NT-proBNP was included in the statistical model. This suggests that there may be additional mechanisms underlying elevated hs-cTn-I in RA, compared to those that drive NT-proBNP.

Patients with RA have an increased risk of ischemic heart disease. Thus, pre-existing heart disease could account for differences between RA patients and controls. Indeed, we found a significant correlation between hs-cTn-I concentrations and CACS, a measure of coronary artery atherosclerosis. However, the association between hs-cTn-I concentrations and CACS was not significant after adjustment for age, sex, race, and Framingham risk score. Furthermore, when we adjusted for cardiovascular risk factors, hs-cTn-I concentrations remained significantly higher in patients with RA than controls.

These observations suggest that the clinical and subclinical measures of heart disease we studied do not fully account for the increased concentrations of hs-cTn-I concentrations in RA patients. The most plausible explanation is that elevated hs-cTn-I concentrations in RA patients reflect subclinical myocardial damage, perhaps reflected by small vessel disease or fibrosis, in addition to that accrued from hypertension, age, and other cardiovascular risk factors.

Another consideration is whether the increased concentrations of hs-cTn-I in RA could be artifactual since rheumatoid factor and other heterophilic antibodies can potentially interfere with antibody-based immunoassays by nonspecific binding with detection agents. Rheumatoid factor, present in 71% of patients in this study, however, was not associated with higher circulating hs-cTn-I. Furthermore, in developing the assay, we designed the assay buffers to contain blockers to suppress interference from heterophile antibodies, including RF and human anti-mouse antibodies. The assay was tested experimentally with samples known to contain high concentrations of such interfering substances and no interference was observed.

A limitation of the study was that we did not have measures of myocardial structure or function. Furthermore, the study was cross-sectional, and thus provided no information about long-term outcomes. We could not evaluate the temporal relationship between inflammatory markers and troponin. We cannot, for example, exclude the possibility that waxing and waning concentrations of inflammatory mediators during exacerbations of RA may affect hs-cTn-I concentrations. The patients in this study had relatively well-controlled disease and it is possible that high levels of inflammation, as occurs in poorly controlled disease, may affect hs-cTn-I concentrations.

In conclusion, high-sensitivity cTn-I concentrations are elevated in patients with RA without heart failure, independent of cardiovascular risk profile and inflammatory markers. Elevated troponin concentrations in RA may indicate subclinical, indolent myocardial injury.
